# Bonitation assessment of intensively used football turf depending on the date and area of observation

**DOI:** 10.1038/s41598-023-42562-8

**Published:** 2023-09-20

**Authors:** Karol Wolski, Łukasz Sobol, Henryk Bujak

**Affiliations:** 1 Institute of Agroecology and Plant Production, Wroclaw University of Environmental and Life Sciences, Grunwaldzki Sq. 24A, 50-363 Wroclaw, Poland; 2 Department of Applied Bioeconomy, Wroclaw University of Environmental and Life Sciences, Chełmońskiego St. 37a, 51-630 Wroclaw, Poland; 3 Department of Genetics, Plant Breeding and Seed Production, Wroclaw University of Environmental and Life Sciences, Grunwaldzki 24A, 50-363 Wroclaw, Poland; 4 Research Centre for Cultivar Testing, Słupia Wielka 34, 63-022 Słupia Wielka, Poland

**Keywords:** Agroecology, Grassland ecology, Agroecology

## Abstract

This article aimed to evaluate the visual and functional characteristics of intensively used football turf over 10 years, depending on the different areas of the game. The research was conducted on the football turf of the AZS Environmental Club in Wrocław (N: 51° 7′ 31′′ E:17° 4′ 14′′). High variability of the evaluated parameters was observed regarding seasonality, year of observation, and the area of play. It has been shown that the goal area and penalty box areas have the lowest functional value, which are vital areas of the game from the point of view of gaining an advantage in the game. Also, these places are more susceptible to creating sites without plants (requiring additional overseeding) due to the potential of hollowing and goalkeeper interventions ending with the body landing on the ground. The middle area was characterized by the highest overall aspect, color, and turf density values. In the vast majority of cases, there was a downward trend in the turfs’ functional value with the turf’s age, which is an essential finding in the context of the use of intensively used, athletic natural grass surfaces.

## Introduction

The development of physical culture in the modern world is a civilization, cultural and social value^1–3^. Physical activity improves the quality of social and economic life and constitutes the foundation for human capital development^4,5,6^. Sports infrastructure is one of the most dynamically developing markets in Poland and the world^7,8^. However, the relationship between the frequency of use of sports infrastructure and its wear and tear over long periods is still an unsolved problem.

The proper condition of the natural grass surface ensures safety and quality of use^9–11^, and increases the spectacularity and viewership of sports struggles^12–15^. The grass turf is a natural barrier between the player and the ground, it absorbs the turns and falls of the players during the game^14,16–18^. A firm, dense, durable grass surface is an even surface that creates total turf and a dark green color^19,20^. Pratotechnical treatments carried out correctly and on time ensure the high usable quality of the turf^21–23^. The overall aspect (Ao), turf density (D), and color (C) of the football turf depend on the selection of grass species and varieties with appropriate compositional homogeneity in turfgrass mixtures^22,24,25^. Meadow grass *Poa pratensis* L., perennial ryegrass *Lolium perenne* L., and red fescue *Festuca rubra* L. play a key role in creating natural grass surfaces^14,22,25–30^. Specialized varieties allow you to achieve the best results^31,32^. *P. pratensis* creates a permanent branch- loose structure, dense turf, emerald green, and high turf-forming capacity^30^. It is resistant to intensive exploitation, low and frequent defoliation (mowing) and nutrient deficiencies^33–35^. On the other hand*, L. perenne* has a loose structure, has a high tolerance to intensive use, and is characterized by quick self-reconstruction^36^. *F. rubra* creates the following forms: creeping—ssp. genuina Hack. and semi-runners—ssp. trichophylla Gaud., which are used for sports athletic grounds^26,37^.

In the United States and Europe, football turfs are assessed according to the National Turfgrass Evaluation Program (NTEP)^38^, while in Poland, according to the system of the Research Centre for Cultivar Testing (COBORU)^39^. These systems are based on the classification and assessment of appropriate parameters (i.e. overall aspect of turf, turf density, turf color), allowing to obtain turf with proper aesthetic and functional values (usefulness in the game).

One of the critical factors shaping the visual and functional aspects of football turf is the intensity of its use. To maintain the high-quality natural grass sports field already at the construction stage, base grading, material selection, subsurface drainage, irrigation installation, sand type, and appropriate species of grass mixture should be selected^40^. This is particularly important since natural grass surfaces used in open stadiums and sports and recreation complexes are strongly influenced by seasonal and daily weather changes, intensive prato-and agrotechnical maintenance (renovation, prevention of damage), dewatering, and wear response^41–45^. Compared to artificial grass turf, which allows for relatively greater intensity of use (up to 50 hours a week) and durability in all weather conditions^46^, a natural grass surface is more susceptible to damage (assuming the same time of use of the turf). Our team's previous research has shown that after ten years of using intensively exploited natural sports surfaces, turfs are partially degraded. Mainly it is related to the reduction of the leaf surface, which in turn leads to the inhibition of grass growth—especially the root system, the range of which reduces up to 6 cm on average^47^. It is worth emphasizing, however, that the changes in the functional and visual characteristics of the football ground do not run uniformly over the entire playing field area^47^. This is because footballers strategically select game zones in which the turf is used more intensively, e.g. to perform attacks in which an advantage is obtained, leading to the creation of a chance to score^48^ or search for a zone a free space^49^ into which the introduction of the ball will be associated with gaining an advantage or an advantageous position for the pass or shot. In other words, there is significant wear and tear on the sports surface in some areas due to heavy traffic near the goal. The surface hardness observation conducted by Miller^50^ also showed that within football fields, there are spatial relations on units built of native soil or sand-based substrate. Admittedly, the earlier mentioned experiment^50^ was not designed to determine what factors might have influenced the surface hardness of football pitch, but it did identify some characteristic points that could indicate essential areas of the playing field where the surface changes have occurred.

However, in the current literature, there is very little information about changes in the turf’s characteristics and visual- functional characteristics in the football field’s various playing zones. Additionally, most research focuses on determining short-term (up to three seasons) or long-term effects, showing only differences between the first and last year of observation.

In this case, it is challenging to determine what changes took place between years of use and determine the turf’s rate of degradation or regeneration. In addition, prolonged observation of intensively used turfgrass broken down by specific game areas may help to understand the wear of athletic grass surfaces and other ecosystems that require many prato- and agrotechnical treatments. Studies conducted over a more extended period may identify hot spots susceptible to a decrease in functional and visual value and indicate the period followed by a rapid decline in turf quality. This information may be necessary to efficiently manage the turfgrass system and adequately protect critical areas of the turf, avoiding the risk of replacing it.

The purpose of this study is to analyze the visual and functional features (general aspect, density, color, and turf functional value) of football turf over ten years of use, de-pending on the typical zones of the football field.

## Materials and methods

### Study site

Prolonged observation of the turf was conducted on a natural, grassy turf belonging to the AZS Environmental Club in Wroclaw, Poland, in the GEM sport complex, which is a residential complex of the Polish women's national football team. The location details of the turf are included in Table 1.

**Table 1:** Localization parameters of observed turf.

Parameter	Description
Object coordinates	N: 51° 7′ 31′′ E: 17° 4′ 14′′
Climate	Temperate climate zone
Watercourses	On the banks of Odra River
Landscape location	In the Szczytnicki nature and landscape complex

### Turf characteristics

The football turf was established in 2002, consisting of 3 turf types of grass in the following proportion: *Lolium perenne* 40%; *Poa pratensis* 40%; *Festuca rubra* 20%. Turf parameters are included in Table 2.

**Table 2:** Technical parameters of observed turf.

Parameter	Value/description
Vegetation layer	Made of loose, slightly loamy sand
Floatable parts content	9.5%
pH of soil	6.8
Permeability	60 dm^3^ h^−1^
Density	0.75 MPa
Organic carbon content	1.9%

### Frequency of use of football turf

About 15–16 football matches were played on the field during a spring/autumn round (about 30–32 matches on average per whole season). There were 2 training units per week directly before the matches. During training units, the pitch was used evenly across the entire section, without avoiding the involvement of specific sectors of the game.

### Pratotechnical and agrotechnical treatments on football turf

From April to September (2005–2014), the football turf was irrigated, depending on the weather, with a dose of 7–10 dm^3^ m^−2^ (daily). The first mowing was made when turf height reached 7 cm, the grass was mowed to 5 cm. During the league season, turf was mowed 1–2 times a week to a height of 2.8 cm. During the growing season, NPK mineral fertilization was applied in the proportions of 6:2:4 with a nitrogen dose of N: 180 kg ha^−1^; P_2_O_5_: 60 kg ha^−1^; K: 120 kg ha^−1^, using the spring and summer fertilizer NPK 17–6–11 + MgO + S + B and autumn NPK 5–0–25 + S + Ca + Fe + B.

### Bonitation assesment

The research was carried out in 2005–2014, in four replications, taking into account three factors (A) the time of observation, spring and autumn round (A_1–2_), place on the football field (B_0–4_) and years of research (C_1–10_). The COBORU valuation method assessed the football turf (visual observation of the turf). The evaluation with the bonitation method included: general aspect (Ao), density (D), and color (C). The grading scale takes points from 1 to 9. The higher the number, the more desirable the value of the assessed feature. In each year, after the completion of the research, the football turf functional value (Wum) was calculated according to the following formula^51^:

1 $${\text{Wum}} = 0.34 \cdot {\text{A}}_{{\text{O}}} + 0.33 \cdot {\text{D}} + 0.33 \cdot {\text{C}}$$

where: Wum—turf functional value, A_o_—overall aspect of turf, D—turf density, C—turf color.

### Climate and weather conditions at the observation site

Table 3 presents the climate for the city of Wroclaw (Poland), where prolonged observations of the football pitch were carried out. The data is an average of 1991–2021, excluding the hours of sunshine (1999–2019)^52^.

**Table 3:** Climate and weather conditions in Wroclaw^52^.

	Mean temp. (°C)	Min. temp. (°C)	Max. temp (°C)	Precipitation (mm)	Humidity (%)	Rainy days (days)	Sunshine hours (h)
January	− 0.4	− 3	2.1	49	81	8	3.3
February	0.8	− 2.3	3.9	40	79	8	4.2
March	4.3	0.3	8.4	54	75	9	5.7
April	9.9	4.8	14.7	46	67	7	8.7
May	14.7	9.7	19.2	64	67	8	10.2
June	18.2	13.3	22.4	76	66	9	11.1
July	20.1	15.5	24.3	98	67	9	11.2
August	19.9	15.2	24.3	67	66	8	10.5
September	15.3	11.2	19.5	64	71	7	7.6
October	10.4	7	14	48	77	7	5.2
November	5.6	3.1	8.4	46	83	7	3.9
December	1.4	− 0.8	3.7	48	81	8	3.3

### Statistical analysis

Three-way analysis of variance (ANNOVA) was performed to determine the role of factors and their interactions in shaping the utility value of football turf. Because all factors showed a very strong statistical significance (*p* < 0.001), in order to determine the role of the area of observation, a one-way analysis of variance was performed, divided into individual years and dates of observation (autumn, spring)^53^. Post-hoc test performed with Tukey’s HSD test. In order to determine the correlation between the examined visual and functional features of the turf, the correlation coefficient r was determined. Statistical analyzes were performed at the significance level of *p* = 0.05 in the Statistica 13.0 (StatSoft—DELL Software, TX, USA) program.

In order to determine the downward or upward trend of visual-functional features as a function of age, the R^2^ coefficient of determination was calculated for the football turf in Microsoft Excel. The R^2^ coefficient has been determined separately for specific regions of the football pitch (penalty box, goal area, etc.).

## Results

### Main results of statistical analysis

Table 4 shows the probability value (p-value) of three-way ANNOVA for the parameters evaluated in the experiments (overall aspect, density, color, and turf functional value Wum). The analysis shows that all effects (year, season, place of the game) have a statistically significant influence on the parameters evaluated (*p* < 0.05). However, concerning the interaction of effects, their impact is no longer as strong as that of single effects. Admittedly, in the case of the interaction of two parameters, a strong influence on individual parameters can be observed in some cases (e.g. the influence of the interaction of Year and Place on Overall Aspect and turf functional value Wum). However, in the case of the interaction of three parameters, the influence was not statistically significant.

**Table 4:** The probability value (*p* value) of the three-way ANNOVA for the parameters evaluated.

Effect	Overall aspect	Density	Color	*Wum*
Year	0.000	0.000	0.000	0.000
Place	0.000	0.000	0.000	0.000
Season	0.000	0.000	0.000	0.000
Year∙place	0.000	0.233	0.535	0.000
Year∙season	0.409	0.057	0.050	0.000
Place∙season	0.409	0.051	0.771	0.022
Year∙place∙season	0.978	0.921	0.967	0.349

### Overall aspect

**Figure 1:**
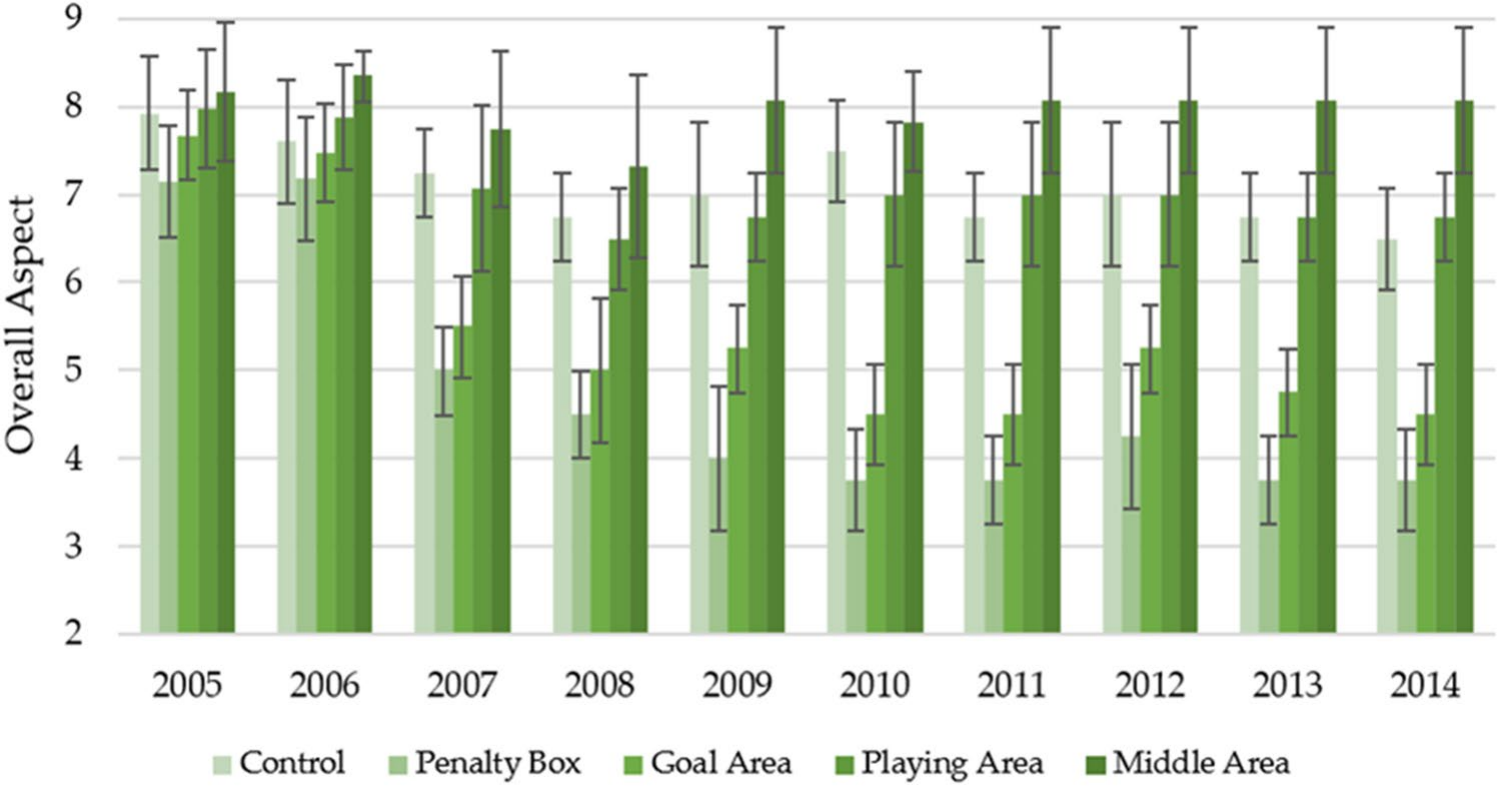
Changes in the overall aspect in spring in individual parts of the football turf field during 10 years of research (2005–2014).

Figure 1 shows changes in the overall aspect of the turf, depending on the area of observation and the year of the observation in the spring. It can be seen that the highest values were found in the middle area, which over ten years, with slight fluctuations, remained around 8. It was basically the only area of observation that did not change its value relatively much, along with the age of the turf. A decreasing trend of the overall aspect was reported for the remaining observation areas, along with the turf age. However, in two cases—this trend was much milder. On average, for the controls, the overall aspect decreased from 7.93 ± 0.64 to 6.50 ± 0.58, while the playing area decreased from 7.98 ± 0.67 to 6.75 ± 0.50. For the penalty box and goal area, the decrease was much more drastic as corresponding decreases from 7.15 ± 0.78 to 3.75 ± 0.50 and 7.68 ± 0.51 to 4.50 ± 0.58 respectively, were observed. It is also worth noting that for these observation sites, an apparent decrease was reported between the second and third years of observation (2006/2007), which was not observed on such a large scale for the other game areas.

**Figure 2:**
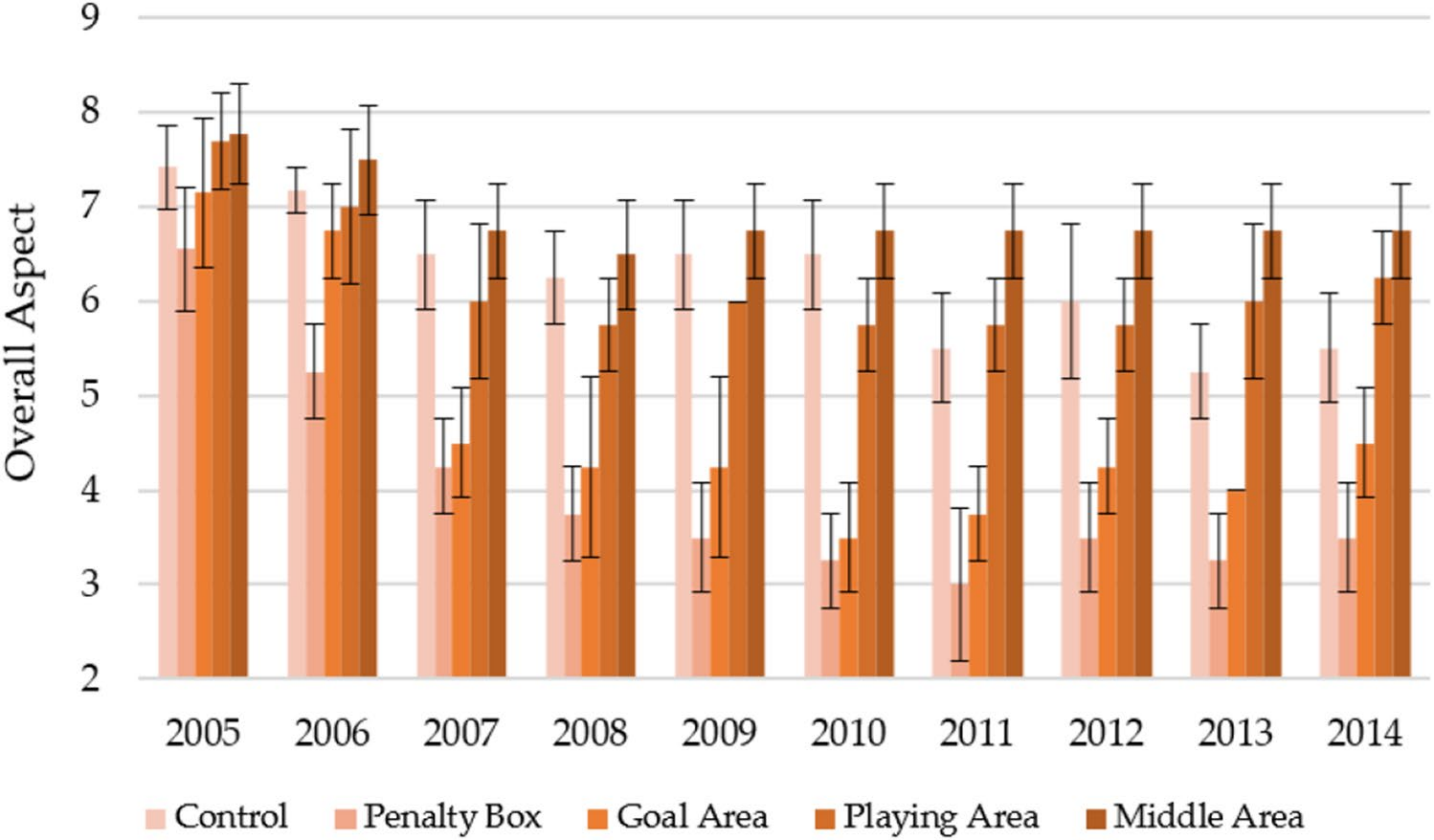
Changes in the overall aspect in autumn in individual parts of the football turf field during 10 years of research (2005–2014).

Similar downward trends were recorded in the autumn season (Fig. 2). However, the overall aspect was characterized by a lower value than spring. On average, over the 10 years of observation, the middle area (6.90 ± 0.60) was characterized by the highest overall aspect, decreasing from 7.78 ± 0.53 in the first year of observation to 6.75 ± 0.50 for the last year of observation. During the research period, the average overall aspect control and playing area level were similar and amounted to 6.26 ± 0.84 and 6.20 ± 0.81, respectively. Similarly to the spring season, the lowest overall aspect control values were found for the penalty box, which was 3.98 ± 1.18 over 10 years (with a decrease from 6.55 ± 0.66 to 3.50 ± 0.58 between the first and last years of use). The goal area, which on average was at the 4.69 ± 1.32 level, showed a slightly higher parameter value, recording a decrease from 7.15 ± 0.79 to 4.50 ± 0.58 between the first and last years of observation.

Table 5 shows the statistically significant changes in overall aspect during the first and last year of observation and, on average, at the turn of the decade. It can be seen that both in the spring season and in the autumn season, for the first year of observation, the game sites did not show statistically significant differences. They began to be classified later, along with turf age. Hence, it can be noticed that both in the last season of the game and on average over 10 years, the places of the game were statistically different in terms of the overall aspect of the turfs, both for the spring and autumn seasons.

**Table 5:** Statistically significant changes in the overall aspect during the first year of play, last year of play, and decade of play.

Part of field		First year of play (2005)	Last year of play (2014)	Decade of play (2005–2014)
Spring	Control	7.93a ± 0.64	6.50b ± 0.58	7.10a ± 0.70
	Penalty box	7.15a ± 0.78	3.75a ± 0.50	4.71b ± 1.40
	Goal area	7.68a ± 0.51	4.50a ± 0.58	5.44c ± 1.24
	Playing area	7.98a ± 0.67	6.75bc ± 0.50	7.07a ± 0.76
	Middle area	8.18a ± 0.79	8.08c ± 0.83	7.98d ± 0.75
Autumn	Control	7.43a ± 0.44	5.50bc ± 0.58	6.26a ± 0.84
	Penalty box	6.55a ± 0.66	3.50a ± 0.58	3.98b ± 1.18
	Goal area	7.15a ± 0.79	4.50ab ± 0.58	4.69c ± 1.32
	Playing area	7.70a ± 0.51	6.25 cd ± 0.50	6.20a ± 0.81
	Middle area	7.78a ± 0.53	6.75d ± 0.50	6.90d ± 0.60
*F *value	Spring	1.324	34.884	70.178
	Autumn	2.737	26.917	61.468
*p* value	Spring	0.306	0.000	0.000
	Autumn	0.061	0.000	0.000

The same marking in column (a, b, c, d, e) indicate no statistical differences (*p* = 0.05) according to Tukey HSD test (in season).

### Density

**Figure 3:**
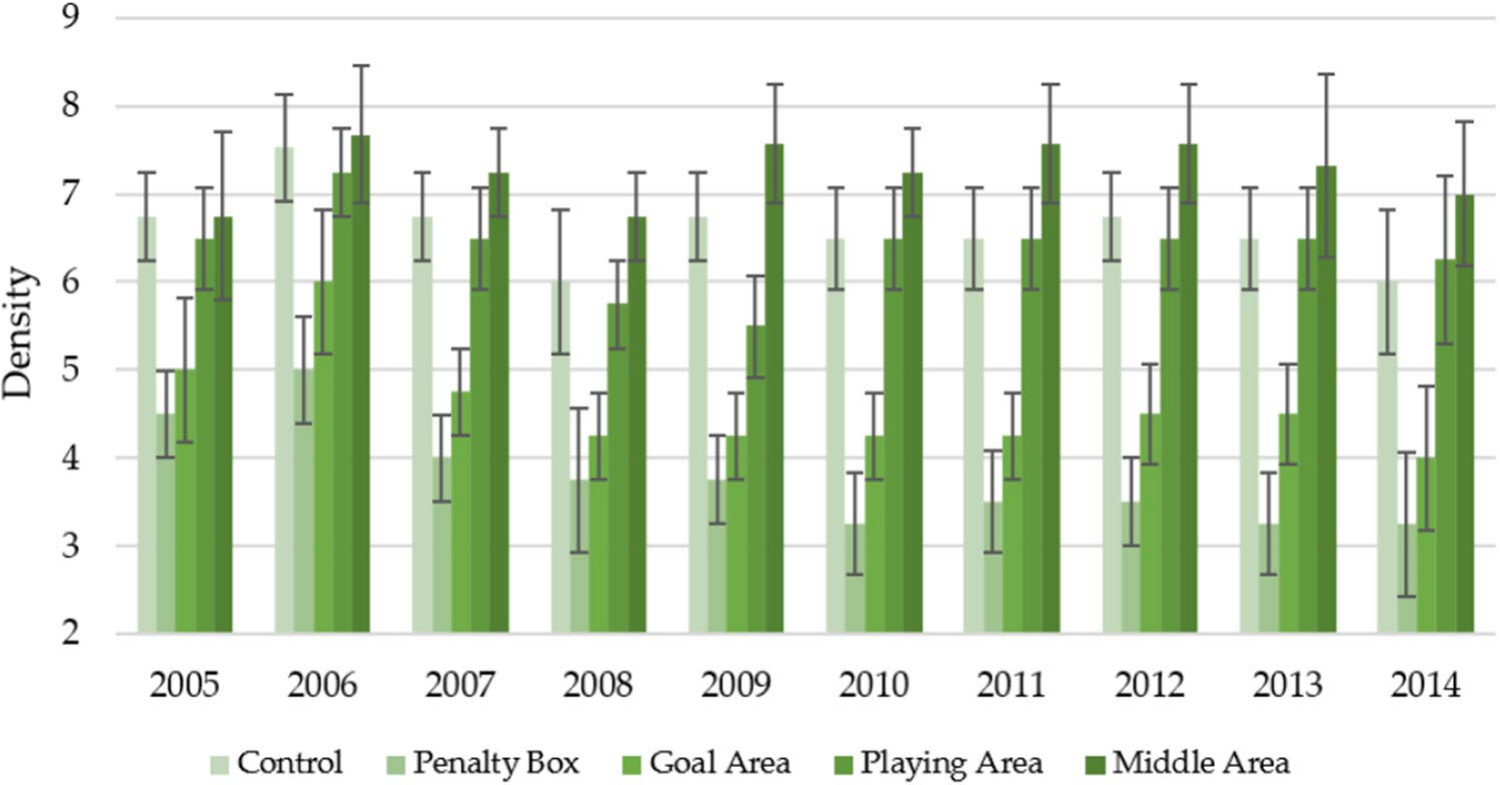
Changes in the density in spring in individual parts of the football turf field during 10 years of research (2005–2014).

Figure 3 shows the changes in turf density over the 10 years of the study, depending on the area of observation in the spring. In most cases, parameter fluctuations were noted without a clear downward/upward trend with increasing turf age. As in the case of the overall aspect, over the years, the middle area was the place with the most attractive density (7.27 ± 0.72). Similar characteristics of changes were also observed for the control and playing area, for which the average density was slightly less attractive compared to the middle area—6.60 ± 0.68 and 6.38 ± 0.70, respectively. For the two areas with the lowest turf density (penalty box and goal area), it was observed that in the later years of turf use, the density is weaker, but in the case of the overall aspect. On average, during the observation, these areas were characterized by a density at the 3.78 ± 0.73 and 4.58 ± 0.78 levels—for the penalty box and goal area, respectively.

**Figure 4:**
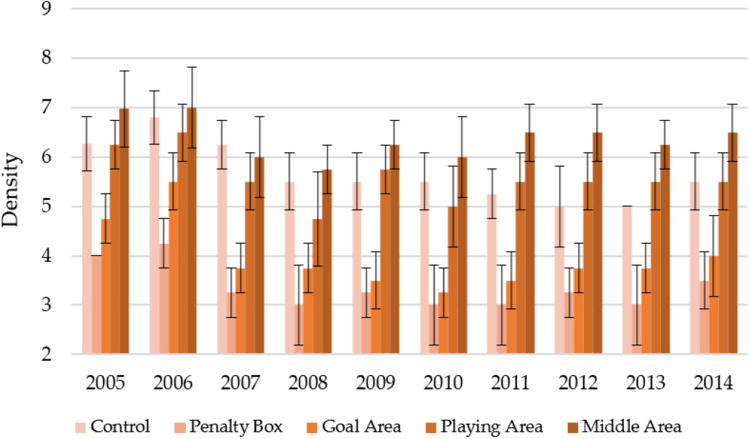
Changes in the density in autumn in individual parts of the football turf field during 10 years of research (2005–2014).

In autumn, turf density was slightly lower than in spring (Fig. 4). However, the characteristics of the differences were similarly preserved as in the case of spring. On average, turf density over the years concerning the classification observation areas was as follows: middle area (6.37 ± 0.70) > control (5.66 ± 0.75) > playing area (5.58 ± 0.85) > goal area (3.95 ± 0.81) > penalty box (3.35 ± 0.70). It was only observed that compared to spring, in the autumn seasons 2012–2014, the goal area and penalty box penalties increased, not consistently decreasing with turf age.

Table 6 shows the statistically significant changes between the game field areas in the first and last years of the game and over 10 years. It can be noticed that statistically significant differences between some cases (control—penalty box/goal area) occurred already in the first year of observation, both for the fall and spring seasons. In the last year of observation, the same classification of homogeneous groups was observed for both seasons. It is worth emphasizing that there is a statistical difference between the middle area and other areas over the 10 years of observation, which proves that this area was dominated by turfing, among other areas of observation.

**Table 6:** Statistically significant changes in the density during the first year of play, last year of play, and decade of play.

Part of field		First year of play (2005)	Last year of play (2014)	Decade of play (2005–2014)
Spring	Control	6.75a ± 0.50	6.00a ± 0.82	6.60a ± 0.68
	Penalty box	4.50b ± 0.58	3.25b ± 0.50	3.78b ± 0.73
	Goal area	5.00bc ± 0.82	4.00b ± 0.82	4.58c ± 0.78
	Playing area	6.50ac ± 0.58	6.25a ± 0.96	6.38a ± 0.70
	Middle area	6.75a ± 0.96	7.00a ± 0.82	7.27d ± 0.72
Autumn	Control	6.28a ± 0.55	5.50a ± 0.58	5.66a ± 0.75
	Penalty box	4.00b ± 0.00	3.50b ± 0.58	3.35b ± 0.70
	Goal area	4.75b ± 0.50	4.00b ± 0.82	3.95c ± 0.81
	Playing area	6.25a ± 0.50	5.50a ± 0.58	5.58a ± 0.75
	Middle area	6.98a ± 0.78	6.50a ± 0.58	6.37d ± 0.70
*F-value*	Spring	9.15	20.66	166.92
	Autumn	21.49	19.50	116.11
*p-value*	Spring	0.001	0.000	0.000
	Autumn	0.000	0.000	0.000

The same marking in column (a, b, c, d, e) indicate no statistical differences (*p* = 0.05) according to Tukey HSD test (in season).

### Color

**Figure 5:**
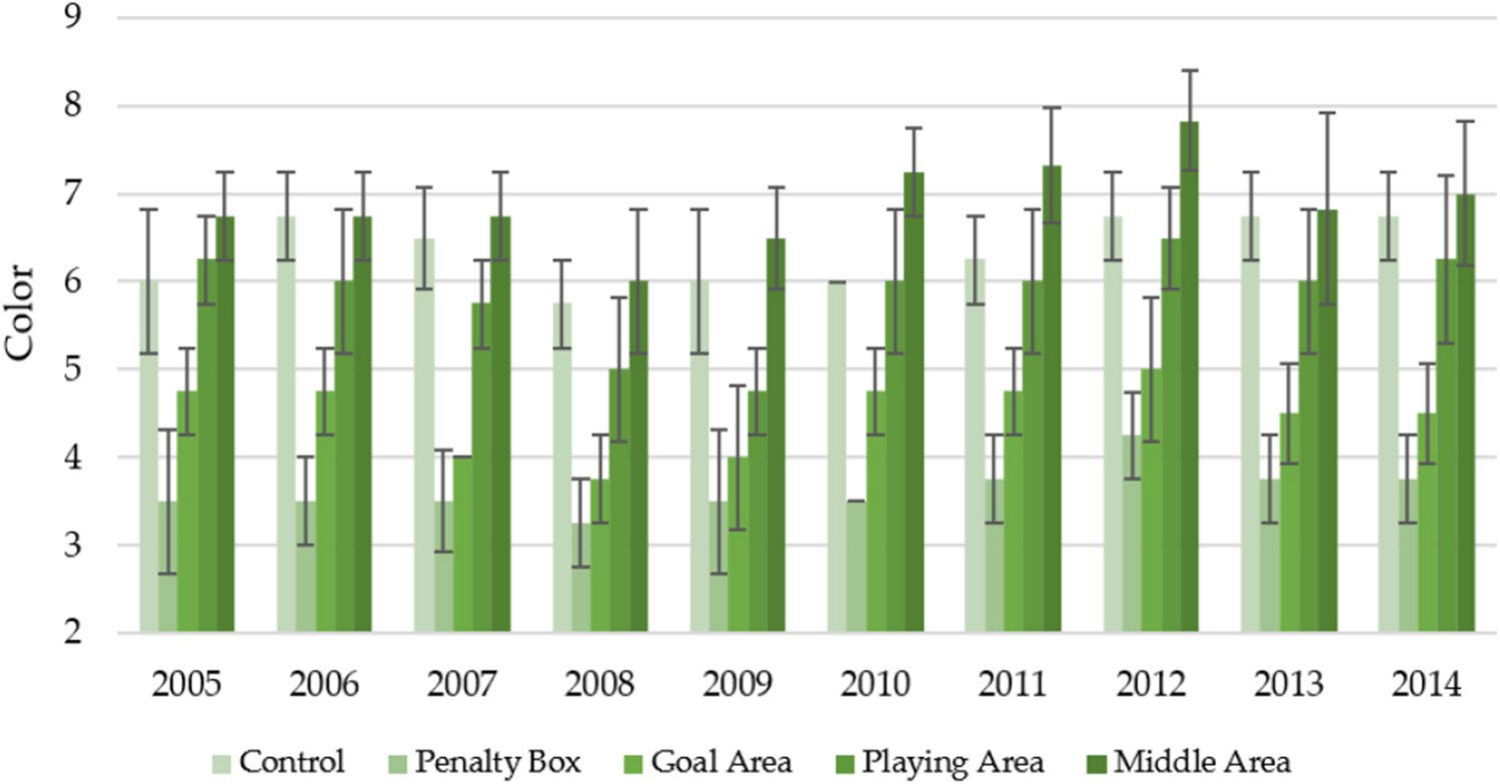
Changes in the color in spring in individual parts of the football turf field during 10 years of research (2005–2014).

Figure 5 shows the color of turfs with a division into observation areas during 10 years of their use in the spring. The most attractive color was that of the middle area, which on average over 10 years had the value of the parameter C = 6.90 ± 0.76. This area's most attractive color was noted for 2010–2014 (6.83–7.83). A slightly less attractive color was observed for the control (average 6.35 ± 0.62) and playing area (5.85 ± 0.83). The areas with the least attractive color were the penalty box and goal area, which on average during the experiment, were characterized by the parameters C = 3.63 ± 0.59 and 4.48 ± 0.64. In-terestingly, the least attractive color for these two areas was observed in 2007–2008 (3.25–3.50 for the penalty box, 3.75–4.00 for the goal area).

**Figure 6:**
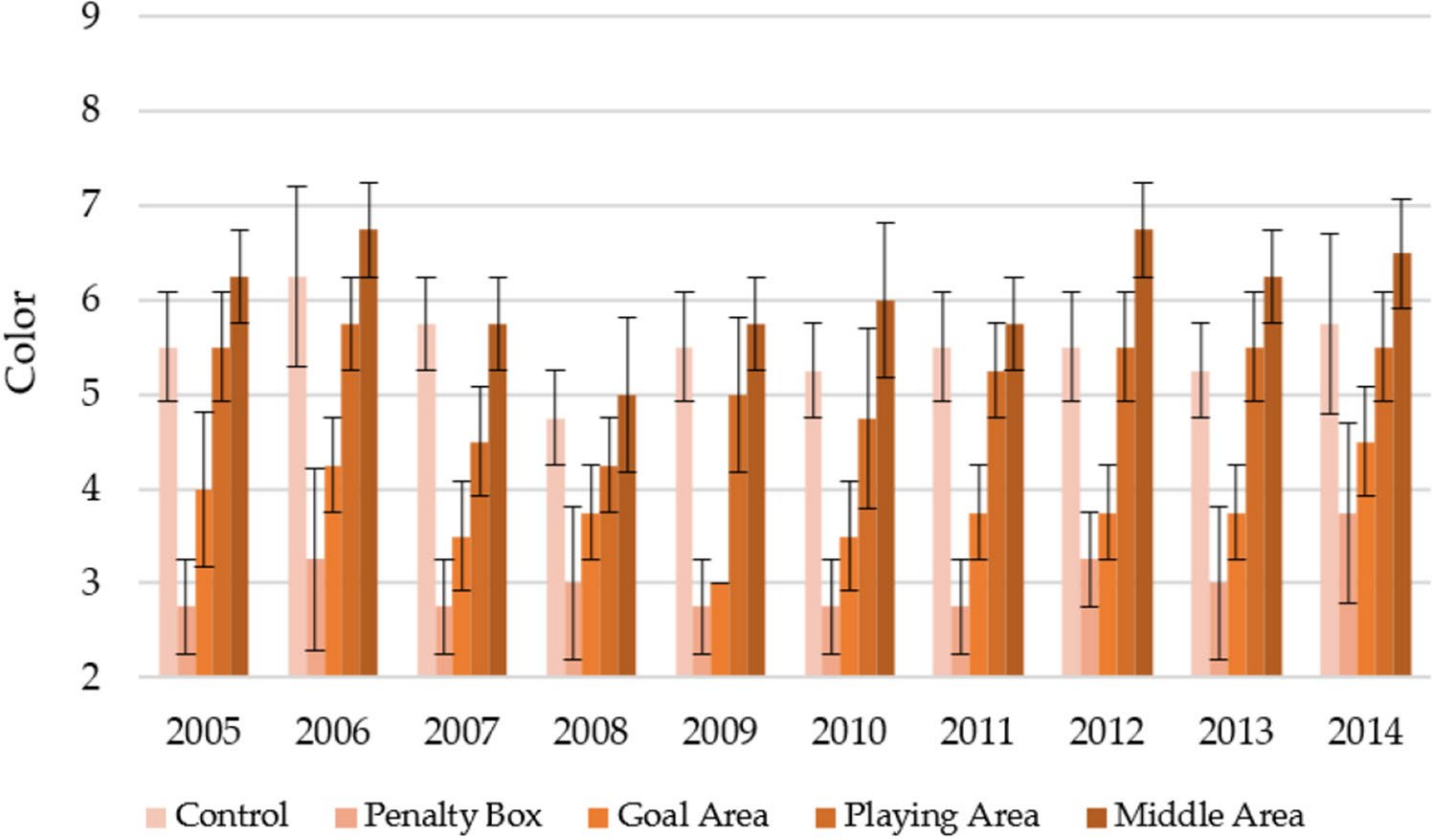
Changes in the color in autumn in individual parts of the football turf field during 10 years of research (2005–2014).

The color of the turfs for the autumn season was much less attractive than for the spring season (Fig. 6). The middle area over the 10 years of observation was characterized by the most attractive color (6.08 ± 0.73). Slightly lower values were noted for control (5.50 ± 0.68) and the playing area (5.15 ± 0.74), and the lowest for the goal area (3.78 ± 0.62) and penalty box (3.00 ± 0.68). Compared to the spring season, most observed areas were characterized by the most attractive color in 2006 and 2014. There were also no clear downward/upward trends in the turf color and the increase in the turf age.

Table 7 shows the statistically significant changes in turf color between the areas of the game in the first and last years of the game and over 10 years. It can be seen that both in the first year, the last year, and the entire study period, there were statistically significant differences between the observation sites. It should be emphasized that Penalty Box had the worst least attractive color (statistically significantly) in the spring and autumn season).

**Table 7:** Statistically significant changes in the color during the first year of play, last year of play, and decade of play.

Part of field		First year of play (2005)	Last year of play (2014)	Decade of play (2005–2014)
Spring	Control	6.00ac ± 0.82	6.75a ± 0.50	6.35d ± 0.62
	Penalty box	3.50b ± 0.50	3.75b ± 0.50	3.63a ± 0.59
	Goal area	4.75bc ± 0.50	4.50b ± 0.58	4.48b ± 0.64
	Playing area	6.25a ± 0.50	6.25a ± 0.96	5.85c ± 0.83
	Middle area	6.75a ± 0.50	7.00a ± 0.82	6.90e ± 0.76
Autumn	Control	5.50a ± 0.58	5.75ab ± 0.96	5.50a ± 0.68
	Penalty box	2.75b ± 0.50	3.75c ± 0.96	3.00b ± 0.68
	Goal area	4.00b ± 0.82	4.50ac ± 0.58	3.78c ± 0.62
	Playing area	5.50a ± 0.58	5.50ab ± 0.56	5.15a ± 0.74
	Middle area	6.25a ± 0.50	6.50b ± 0.58	6.08d ± 0.73
*F* value	Spring	19.79	22.33	147.67
	Autumn	21.61	10.72	129.22
*p* value	Spring	0.000	0.000	0.000
	Autumn	0.000	0.000	0.000

The same marking in column (a, b, c, d, e) indicate no statistical differences (*p* = 0.05) according to Tukey HSD test (in season).

### Turf functional value (Wum)

**Figure 7:**
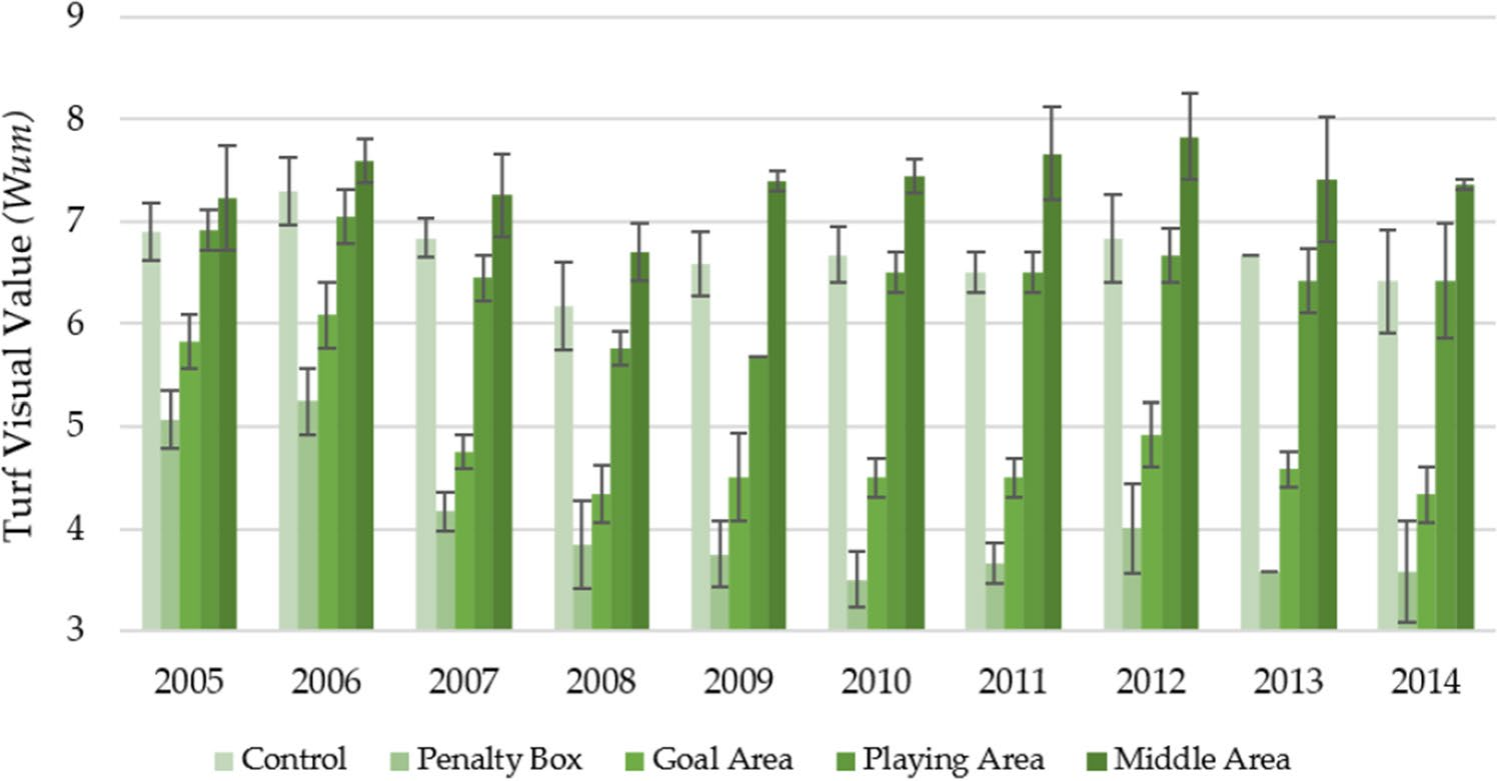
Changes in the turf functional value (Wum) in spring in individual parts of the football turf field during 10 years of research (2005–2014).

Figure 7 shows the changes in the functional value of the turfs depending on the year and area of observation over 10 years in spring. On average, the middle area was characterized by the most significant visual value (7.39 ± 0.43). Along with the increasing turf age (subsequent years of observation), a slightly increasing trend was observed in turf visual value in this area (R^2^ = 0.1179). Slightly lower values of the Wum parameter were obtained for the playing area (6.44 ± 0.48) and control (6.69 ± 0.41). For these areas, a slight downward trend was observed over the years (R^2^ = 0.0395 and 0.2126, respectively). On the other hand, for the areas characterized by the lowest values of the Wum parameter—goal area (4.84 ± 0.64) and penalty box (4.04 ± 0.66), the downward trends were much more pronounced (R^2^ = 0.4503 and 0.6140 respectively).

**Figure 8:**
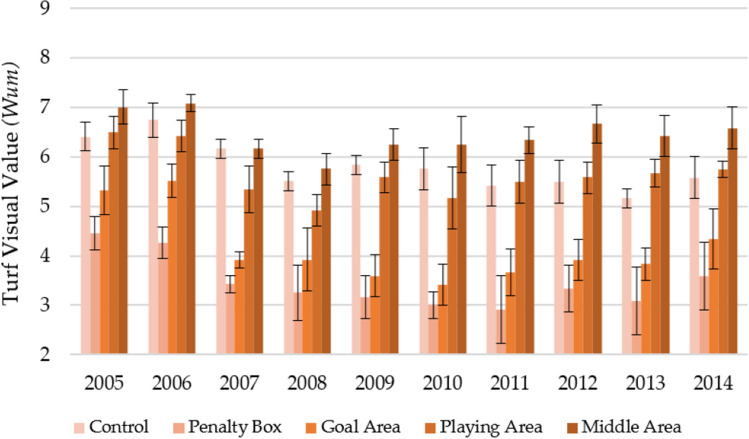
Changes in the turf functional value (Wum) in autumn in individual parts of the football turf field during 10 years of research (2005–2014).

Figure 8 shows the changes in the functional value of the turfs (Wum) depending on the year and area of observation over 10 years in autumn. The functional value of the turfs in the fall was lower than in the spring. Nevertheless, the same classification was kept as in the case of spring: middle area (6.45 ± 0.50) > control (5.81 ± 0.55) > playing area (5.65 ± 0.58) > goal area (3.78 ± 0.62) > penalty box (3.45 ± 0.66). However, it is worth noting the impact of the turf age on the development of the functional value of the turf at individual observation sites. In the case of the middle area and playing area—slight downward trends were observed (R^2^ = 0.0385 and 0.1213, respectively), which may indicate that over the years, this place was characterized by a turf with constant visual parameters. A different situation was observed for the other observation sites for which the value of the Wum parameter showed a downward trend in the subsequent years of observation (R^2^ = 0.3059 for goal area, R^2^ = 0.3908 for penalty box and R^2^ = 0.6859 for control).

It can be observed that both at the beginning and the end of the observation, the observation sites showed statistically significant differences (Table 8). During the 10 years of observation, both in the spring and autumn, most of the observation sites differed statistically from each other. The exceptions were control and playing area, which the same homogeneous group characterized.

**Table 8:** Statistically significant changes in the color during the first year of play, last year of play, and decade of play.

Part of field		First year of play (2005)	Last year of play (2014)	Decade of play (2005–2014)
Spring	Control	6.90a ± 0.28	6.42b ± 0.50	6.69a ± 0.41
	Penalty box	5.07b ± 0.19	3.59a ± 0.17	4.04b ± 0.66
	Goal area	5.83c ± 0.26	4.34a ± 0.27	4.84c ± 0.64
	Playing area	6.92a ± 0.20	6.42b ± 0.56	6.44a ± 0.48
	Middle area	7.23a ± 0.51	7.37d ± 0.05	7.39d ± 0.43
Autumn	Control	6.41a ± 0.29	5.58a ± 0.42	5.81a ± 0.55
	Penalty box	4.45b ± 0.34	3.58b ± 0.69	3.45b ± 0.66
	Goal area	5.32c ± 0.49	4.34b ± 0.61	4.14c ± 0.79
	Playing area	6.50a ± 0.33	5.76a ± 0.16	5.65a ± 0.58
	Middle area	7.01a ± 0.35	6.59a ± 0.42	6.45d ± 0.50
*F* value	Spring	34.10	172.54	262.76
	Autumn	31.65	26.52	155.93
*p* value	Spring	0.000	0.000	0.000
	Autumn	0.000	0.000	0.000

The same marking in column (a, b, c, d, e) indicate no statistical differences (*p* = 0.05) according to Tukey HSD test (in season).

### Relationship between parameters

**Figure 9:**
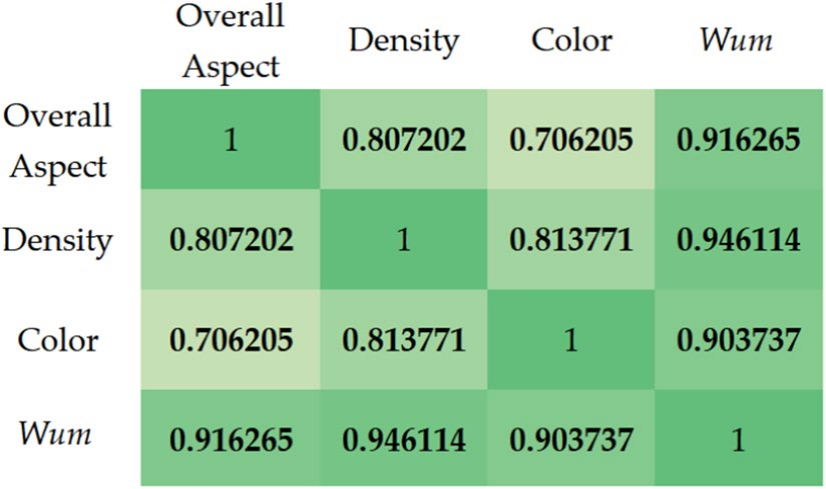
Heatmap for correlation coefficients (r) for evaluated parameters.

Figure 9 shows the values of the correlation coefficients between the parameters included in the turf evaluation. It can be observed that for the evaluated parameters, the obtained coefficients are relatively high (0.706–0.946). The highest values of the coefficients were obtained for the Wum parameter, but it is a component of A_O_, D,and C. Nevertheless, the obtained high rates between A_O_, D and C (0.706–0.814) suggest a high possibility of predicting other parameters with the knowledge of one key factor (Overall Aspect).

## Discussion

The aim of this research was to examine the appearance and performance characteristics of football turf over a period of ten years, focusing on different zones of a football field. The overall perception by players of the quality of the pitch, i.e. its overall appearance (aesthetics often correlated with the actual condition of the field), which has an influence on the comfort of the game, playability, and the risk of injury^54–56^. From the technical point of view, according to Grabowski^57^, based on own and others observations^58–67^, natural turf grass sports fields may vary within the pitch due to diversified player movement, pitch type and its structure, pitch management (irrigation, fertilization, pest and disease control), weather and climatic conditions, grass species, substrate type, fertility, granulometric composition, physicochemical properties, the intensity of use^57–67^. As expected, it was confirmed that the bonitation value of the football turf is highly variable, depending on the year of observation and the examination area. Prolonged observation of the turf can therefore be an exciting tool in determining the period after which there is a rapid decline in the quality of the turf, to select the appropriate date for its renovation. Primarily, it has been observed that the areas with the lowest visual and functional values are the goal area and the penalty box, which is also similar to the results obtained in the previous work^47^ and the experiment conducted by Grabowski et al.^57^. This phenomenon may be related to the fact that these places are mainly exploited because they are critical areas associated with gaining an advantage in the game (increased probability of scoring a goal)^68^.

**Figure 10:**
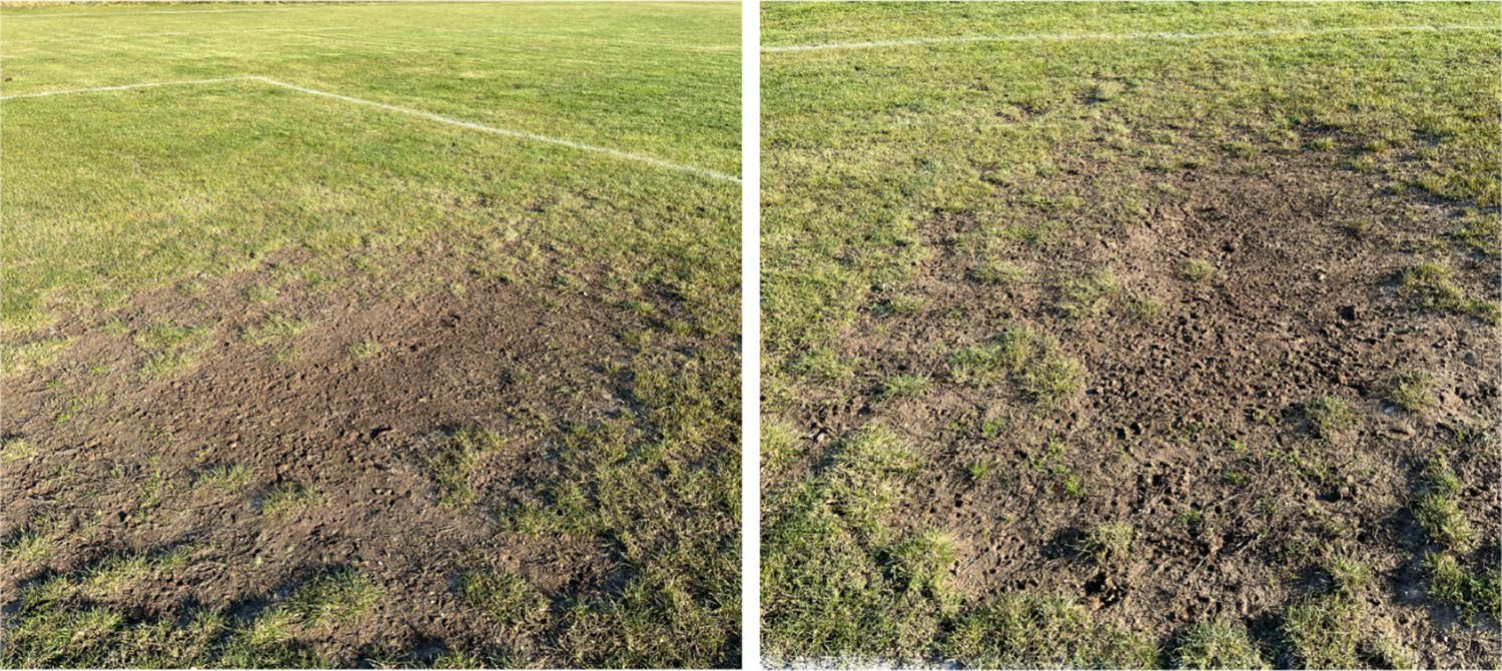
Typical turf damage within the penalty box/goal area due to goalkeeper interventions (own photo).

Additionally, research by Ruiz-Ruiz et al.^69^ showed that entering the penalty area can be considered an indicator of performance, differentiating between winning and losing teams. Hence, the high inrun frequency of both defending and attacking players could cause trampled grass to lose quality, resulting in a deterioration in the density and overall aspect of the turf. These areas (penalty box, goal area) are classified as particularly susceptible to wear and excessive surface damage^70^. In addition, these areas are particularly at risk of creating plantless spots due to goalkeeper interventions (Fig. 10), which result in the body falling to the ground^71,72^ and damaging the surface (requiring overseeding, which is not always possible due to the ongoing league season). As can be seen from the obtained results, it had a lower tendency to deteriorate quality, along with turf age in areas characterized by a higher functional value (middle area, playing area) in the first evaluation periods. It is, therefore, likely that the penalty box and goal area, which tended to deteriorate in their functional value with the intensity of use, may have been damaged (i.e. due to too frequent massive trampling) in the first years of use. The high level of damage could make the grass unable to regenerate and fill the gaps in turf, or due to continued intensive use (league matches, training, tournaments through the year), the grass mixture was not allowed to be torn quickly to fill in spots and damaged marks. However, to confirm this thesis in the future, observations throughout the league season and continuous monitoring of the quality of the turf in every match/training unit are necessary.

Our research also showed high variability of the evaluated parameters (overall aspect, density, color, functional value) in seasonality and years of observation. In general, all parameters tested had a higher value in spring than in autumn, which was also con-firmed in the experiments conducted by Salman et al. and Kir et al.^73,74^. In the context of long-term evaluation of specific turf areas, two cases can be observed: either the functional value of the areas does not change significantly with the aging of the turf, or there is a clear downward trend in the functional value of the turf with the aging of the turf. The first case concerns the middle area and playing area, which were probably areas that were less degraded by the players, which made the grass regenerate more quickly, and the surface could be renovated in a more straightforward way, which allowed for the continuity of similar visual and functional characteristics of the turf. The second case, which occurs mainly for the penalty box and goal area as one of the potential explanations, can probably be explained by the continuing degradation of these areas over the years due to their high importance in the game. Additionally, depending on the composition of grasses, the density of shoots changes significantly over the years. Other research has shown that with age in complex cenoses the density of shoots increases, and in monotypes, it decreases^75^. It is true that initially, a diversified mixture of grasses of various compositions was sown on the evaluated football field (three-species mix). However, previous experiments allowed to conclude that over the years, the share of individual components of lawn mixtures undergoes significant changes compared to the original composition^47^, the maintenance of which is almost impossible. This phenomenon could be especially true for Lolium perenne, which had the highest share in the initial mixture and showed an unfavorable reaction to temperature fluctuations and summer drought^47,76,77^. Hence, the percentage of species that develop less dense shoots may have increased on the turf^75^. The decreasing shoot density, especially in the penalty box and playing area areas, significantly contributed to the deterioration of the functional value of the turf as it correlates with visual turf quality^75,78^. However, to confirm this statement, subsequent research should focus on observing the species composition of the turfs and their changes over many years of examination.

## Conclusion

### Main conclusion of performed observations

The conducted assessment showed that, based on long-term observation (10 years), the visual and functional characteristics of intensively used natural grass turf differ significantly in terms of seasonality, year of observation, and playing field area. The turf with better aesthetic values was noted in the spring seasons.

The penalty box and goal area had the most negligible favorable functional value. They differed significantly in this respect from the middle area and playing field (which showed relatively high values of visual-functional parameters). Such a phenomenon is most likely dictated by the fact that these areas are critical areas in terms of gaining an advantage in the game (frequent runs, tackles, interventions) and have the potential to create areas without plants that require additional overseeding (which is often impossible during the season), due to the possibility of cavities and frequent interventions by the goalkeeper, ending with the body falling onto the turf and damaging the grass surface.

Additionally, in most cases, a downward trend in functional value was observed with the age of the turf. It is an essential find in the context of the use of intensively used natural grass turf in the context of estimating the timing of the field renovation and financial outlays related to maintaining the turf properly.

### Limitations of the research

Despite the standard methodology and parameterization of the results, several factors could have disturbed the long-term observations of the turf proposed in the manuscript. In addition to the complexity of the visual statement of the aesthetics of the turf, it is worth mentioning, among others, the possibility of changing the percentage of grass species and their varieties, which could significantly change over the entire field during a long observation period. In addition, observations of all football matches and training units conducted on the turf were not performed, so the damage could have occurred in other ways than those mentioned in the conclusions and discussion. It is also worth noting that despite the training sessions being conducted across the entire turf, some areas could have been exploited more heavily, making it easier for them to degrade in the match.

### Future recommendations

The manuscript also creates a space for further research in the context of observing long-term changes in the functional and visual characteristics of the natural turfs with a shorter observation interval and determining the changes in the percentage share of grass species and varieties in turf and its impact on the visual and functional characteristics in a specified period. In addition, it is recommended to carefully observe the turf before and after training/match units to determine the degree of improvement in the quality of the turf through standard agro and pratotechnical treatments. Future research should also focus on combining two scientific disciplines (sports and agronomy) to determine the possibility of obtaining and practical use of the correlation between the analysis of the football game and the bonitation value of the turf.

## Data Availability

All data generated or analysed during this study are included in this published article [and its supplementary information files].
